# Supplementary material for: Arginine methylation of METTL14 promotes RNA N6-methyladenosine modification and endoderm differentiation of mouse embryonic stem cells
Source: Nat Commun. 2021 Jun 18;12:3780. doi: 10.1038/s41467-021-24035-6 (PMC8213825; doi:10.1038/s41467-021-24035-6)
Supplement: Supplementary file 1 — Supplementary Information [file 41467_2021_24035_MOESM1_ESM.pdf]

Supplementary Information to

Arginine methylation of METTL14 promotes RNA N^6-methyladenosine modification and endoderm differentiation of mouse embryonic stem cells

Xiaona Liu, Hailong Wang, Xueya Zhao, Qizhi Luo, Qingwen Wang, Kaifen Tan, Zihan Wang, Jia Jiang, Jinru Cui, Enhui Du, Linjian Xia, Wenyi Du, Dahua Chen, Laixin Xia & Shan Xiao

Contents

A Supplementary Figures

B Supplementary Tables

Supplementary Figure 1. METTL14 R255 methylation identification.

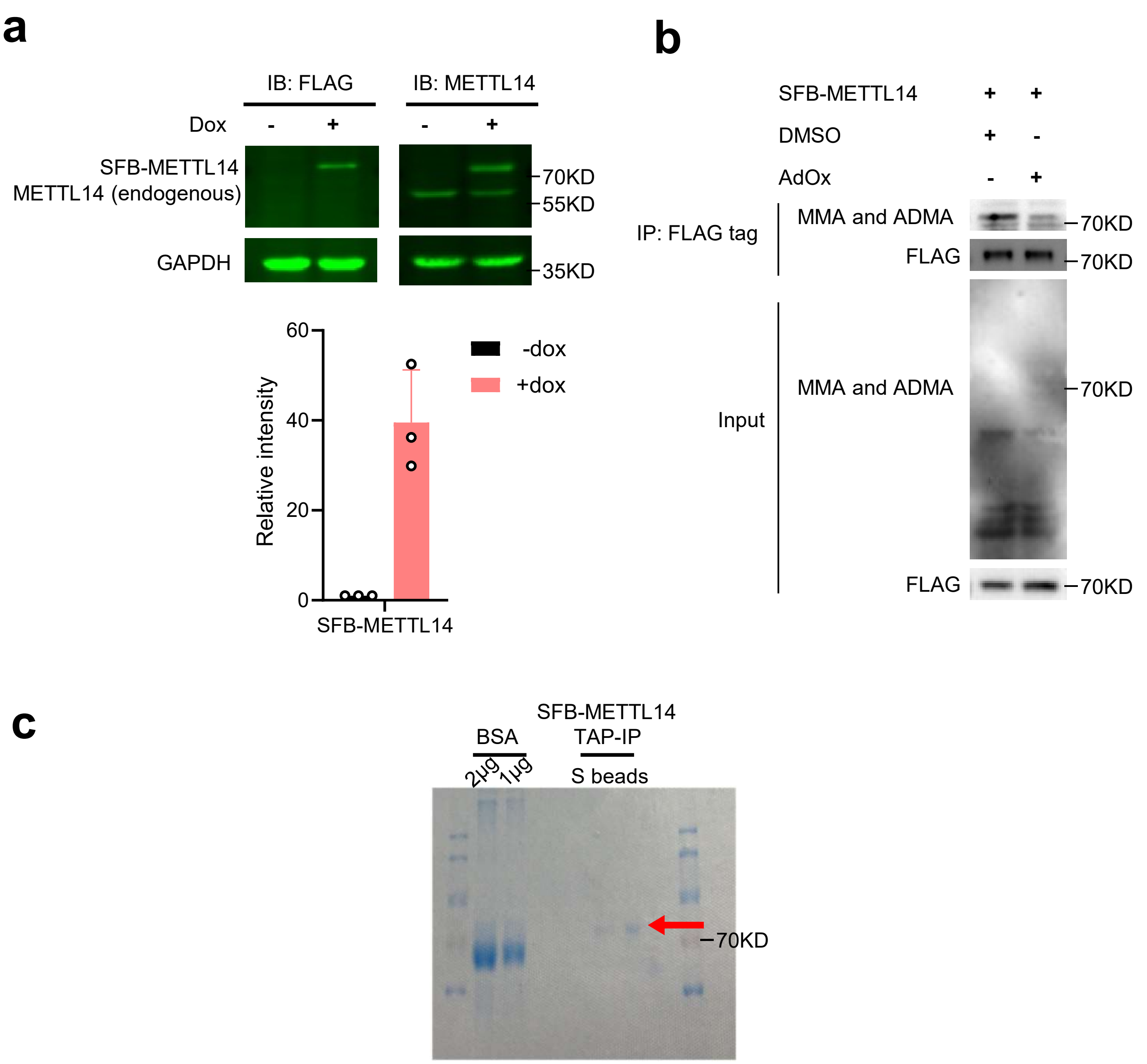

d

Sample	Sequence coverage (%)	Position	Peptide score	Modified sequence	# PSMs	Mass error (ppm)
METTL14	28.73%	Pho S 399	32	LRPKs(ph)PPPK	6	-0.24074
	26.75%	R me255	32	r(me)cEDICWIK	1	-0.26326

e

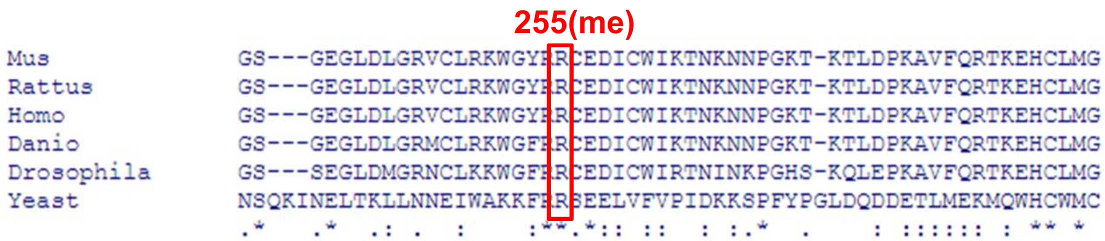

Supplementary Fig. 1. METTL14 R255 methylation identification.

a Near-Infrared (NIR) western blot (top) and quantification (bottom) of METTL14. Ectopic expression of METTL14 was induced by doxycycline treatment of an SFB-METTL14 stable cell line. GAPDH served as a loading control. Representative figures of three independent replicates are shown. Dox, doxycycline. Data are mean ± s.d. of three independent experiments.

b Arginine methylation of immunoprecipitated SFB-METTL14 protein detected by western blot in E14Tg2a cells. Representative figures of two independent replicates are shown.

c Coomassie blue staining of indicated tandem affinity purified SFB-METTL14. The experiment was performed once in HeLa cells. Source data for a-c are provided as a Source Data file.

d Post-translational modifications (PTMs) of METTL14 identified by LC-MS/MS. The sequence coverage (%) was calculated by dividing the number of amino acid sequences identified in LC-MS/MS by the number of amino acids in the full METTL14 sequence.

e Sequence alignment of METTL14 showing conservation of arginine 255.

Supplementary Figure 2. METTL14 R255K ESC line construction and the m⁶A modification features of R255K transcripts.

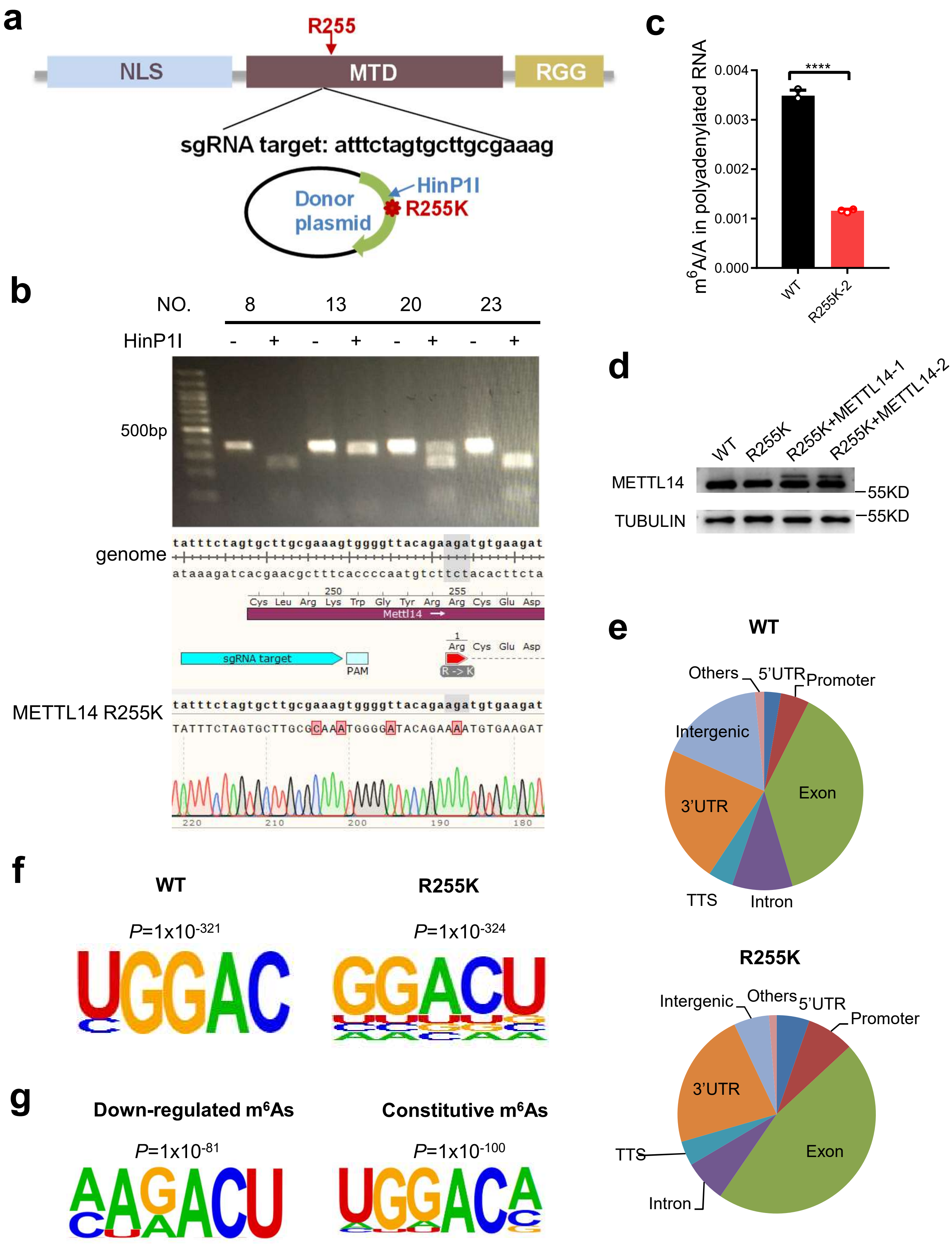

Supplementary Fig. 2. METTL14 R255K mESC line construction and the m^{6}A modification features of R255K transcripts.

a Schematic representation of sgRNA sequences and donor plasmid used to generate METTL14 R255K knock in mES cell line using CRISPR-Cas9. The donor plasmid contains a PCR product of METTL14 genome (green arrow) with HinP1I digestion site and R255K mutations.

b Identification of R255K mESC clones by restriction endonuclease HinP1I digestion (upper) and Sanger sequencing (below). NO.8 and NO.23 are R255K positive clones. Representative figures of three independent replicates are shown.

c LC–MS/MS quantification of m^{6}A abundance in mRNA from a second R255K clone. Data are mean ± s.d. of three independent experiments. Two sided Student's t test, ****$P < 0.0001$.

d Western blot showing the expression level of overexpressed SFB-METTL14 in R255K (the upper band in lane 3 and 4). The lower band is the endogenous METTL14. Representative figures of three independent replicates are shown. Source data for c-d are provided as a Source Data file.

e Distribution of m^{6}A peaks in the 5′ UTR, promoter, exon, intron, TTS, 3′ UTR, and intergenic regions in WT and METTL14 R255K cell lines.

f Sequence motifs identified in m^{6}A peaks in WT and METTL14 R255K cell lines.

g Sequence motifs identified in down-regulated m^{6}A peaks and constitutive m^{6}A peaks in R255K.

Supplementary Figure 3. Prmt1 physically interacts with and methylates METTL14.

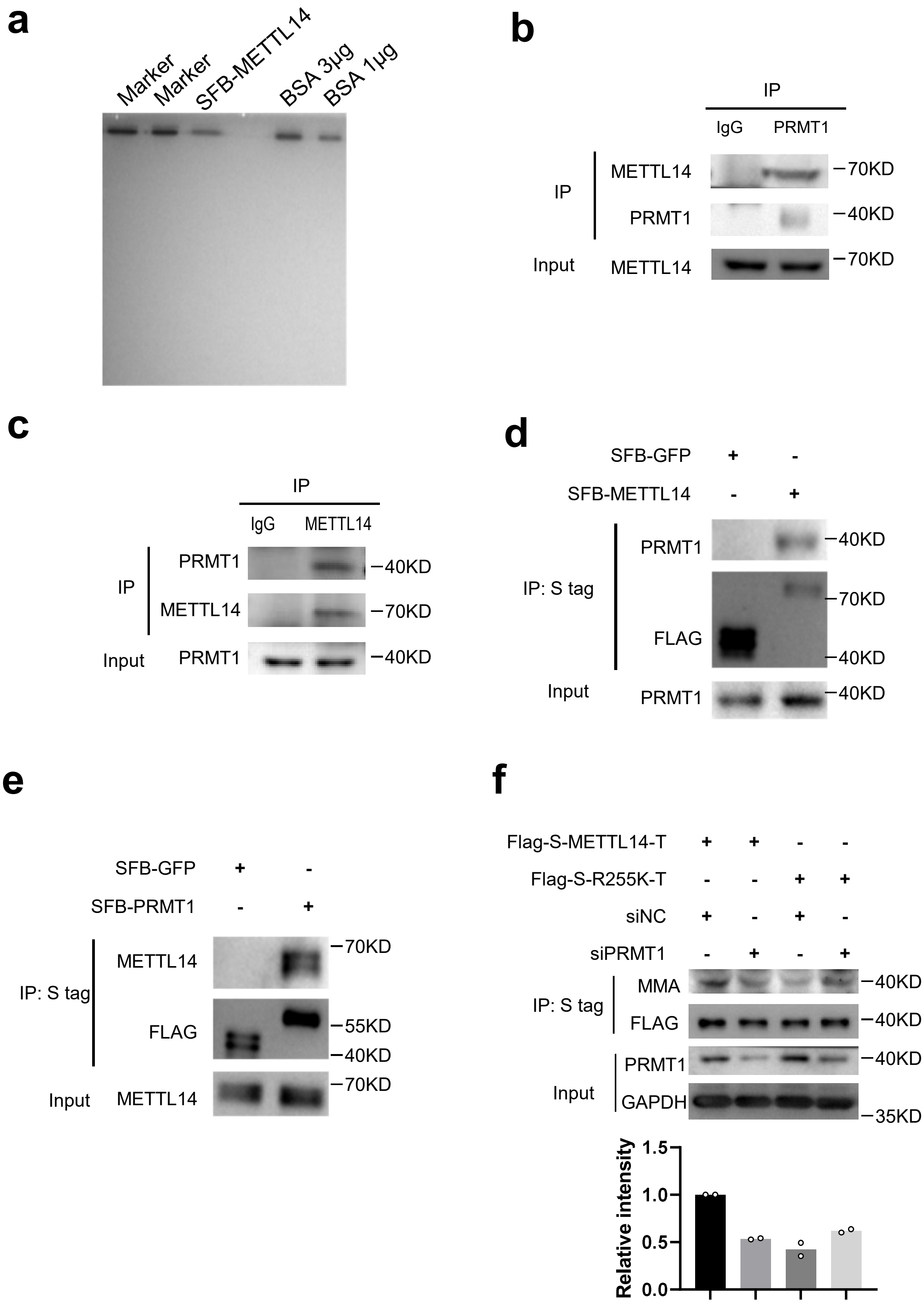

Supplementary Fig. 3. Prmt1 physically interacts and methylates METTL14.

a Western blot showing the interacting proteins with SFB-METTL14 pulled down by tandem affinity purification (TAP), which were subsequently dissected by LC-MS/MS. The experiment was performed once in HeLa cells.

b Co-immunoprecipitation of endogenous PRMT1 with METTL14 in HEK293T cells. The experiment was performed once in HEK293T cells.

c Reciprocal IP of METTL14 with PRMT1 in HEK293T cells. IgG served as negative IP control in (b) and (c). The experiment was performed once in HEK293T cells.

d Co-immunoprecipitation of SFB-tagged METTL14 with endogenous PRMT1 in HeLa cells.

e Reciprocal IP of SFB-tagged PRMT1 with endogenous METTL14 in HeLa cells. For d and e, GFP served as negative control and representative figures of three independent replicates are shown.

f Western blot of mono-methylation of arginine (MMA) on truncated METTL14 and METTL14 R255K (METTL14-T and R255K-T) after *Prmt1* knockdown by siRNA in HeLa cells. siNC, negative control siRNA. Representative figures of two independent replicates are shown. The band intensity of MMA relative to FLAG is shown below, and data from two independent replicates were shown. Source data for b-f are provided as a Source Data file.

Supplementary Figure 4. PRMT1 regulates mRNA m^6A modification.

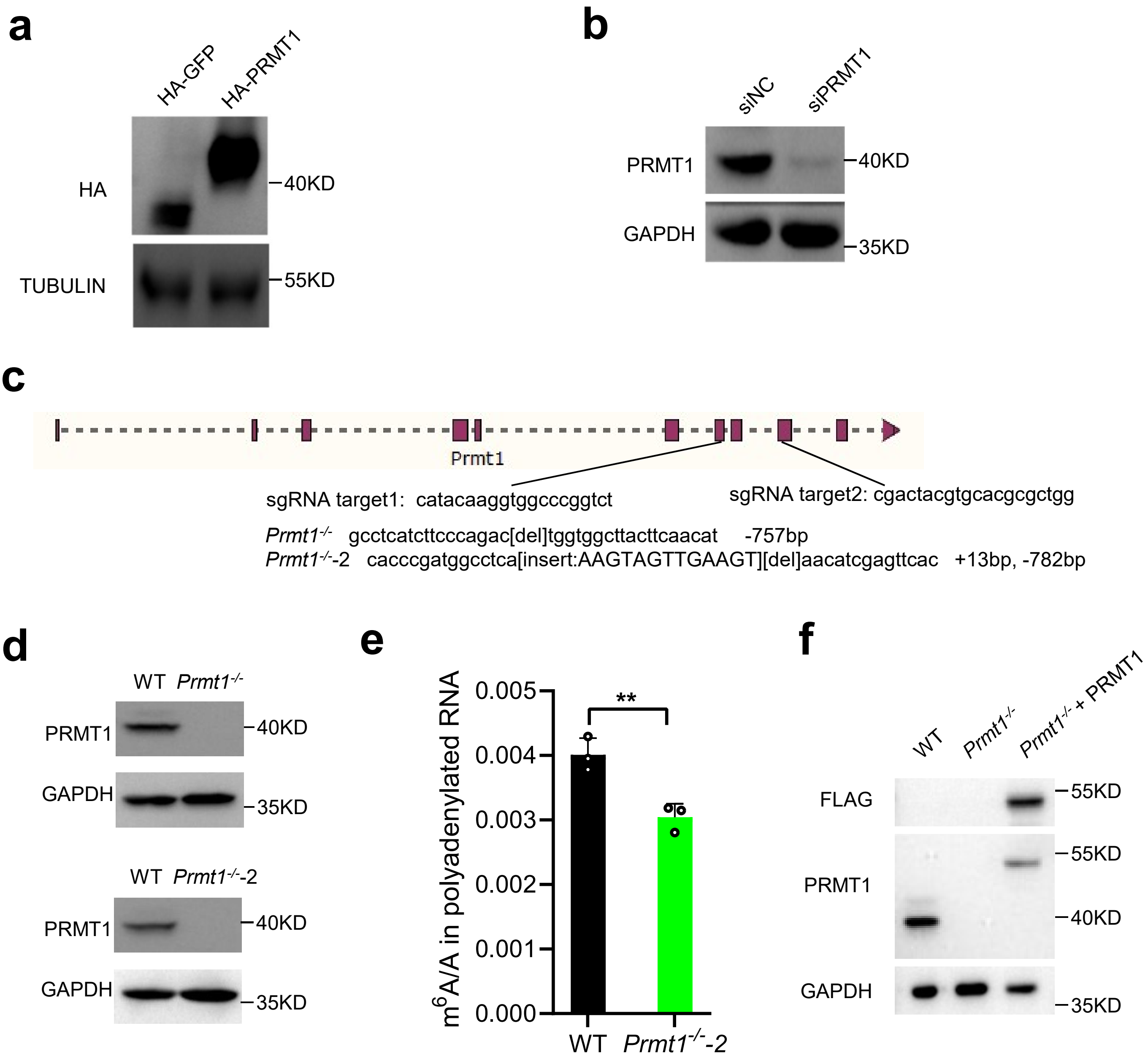

Supplementary Fig. 4. PRMT1 regulates mRNA m^6A modification.

a Western blot of overexpressed GFP or PRMT1. TUBULIN served as control. Representative figures of three independent replicates are shown.

b Western blot of PRMT1 after siRNA knock down. GAPDH served as control. Representative figures of two independent replicates are shown.

c Schematic illustration and sequence of the sgRNAs and mutations of *Prmt1*$^{-/-}$ by CRISPR-Cas9 in mESCs.

d Western blot confirming ablation of PRMT1 in two *Prmt1*$^{-/-}$ mESC clones. Representative figures of three independent replicates are shown.

e LC–MS/MS quantification of m^6A abundance in mRNA from a second *Prmt1*$^{-/-}$ clone. Data are mean ± s.d. of three independent experiments. Two-sided Student's t test. ** $P = 0.0074$.

f Western blot showing the expression level of overexpressed Flag-S tagged PRMT1 in *Prmt1*$^{-/-}$. Representative figures of three independent replicates are shown. Source data for a-b and d-f are provided as a Source Data file.

Supplementary Figure 5. METTL14 R255 methylation stabilizes the methyltransferase complex to its substrate RNA.

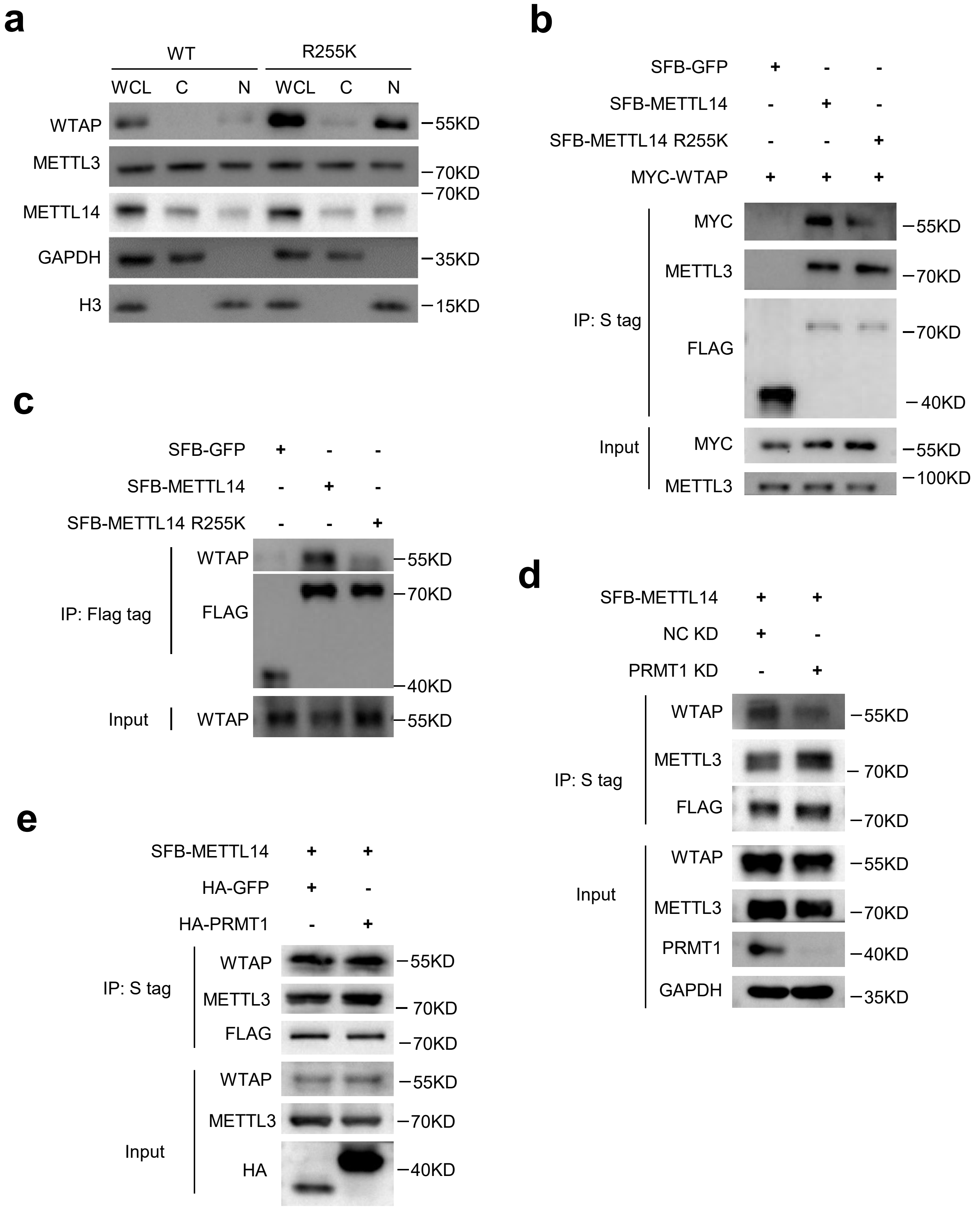

Supplementary Figure 5. METTL14 R255 methylation stabilizes the methyltransferase complex to its substrate RNA.

a Western blot showing the subcellular location of METTL3, METTL14, and WTAP proteins in WT and METTL14 R255K CGR8 cells. WCL, whole cell lysate; C, cytoplasmic fraction; N, nuclear fraction. Representative figures of three independent replicates are shown.

b Co-IP showing the interaction of MYC-WTAP and METTL3 with SFB tagged METTL14 or METTL14 R255K in HEK293T cells. The experiment was performed once in HEK293T cells.

c Co-IP showing the interaction of endogenous WTAP with SFB tagged METTL14 or METTL14 R255K in HeLa cells. The experiment was performed once in HeLa cells. SFB-GFP served as a control in (b) and (c).

d Co-IP showing the interaction of WTAP and METTL3 with METTL14 after PRMT1 knockdown in HeLa cells. Representative figures of two independent replicates are shown.

e Co-IP showing the interaction of WTAP and METTL3 with METTL14 after PRMT1 overexpression in HeLa cells. HA-GFP served as a control. Representative figures of two independent replicates are shown. Source data for a-e are provided as a Source Data file.

Supplementary Figure 6. Molecular simulation shows that METTL14 R255me facilitates the binding with its substrate RNA.

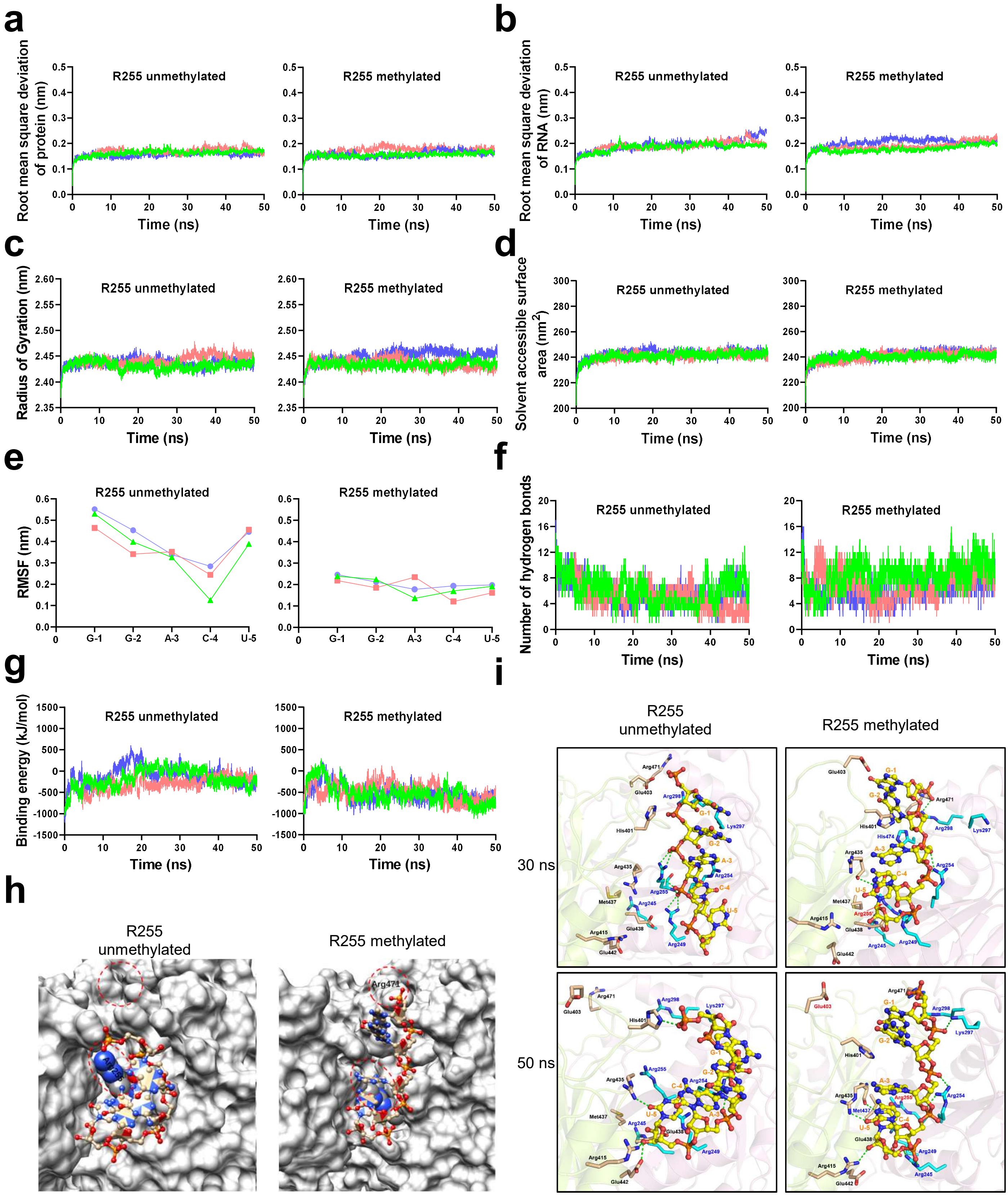

Supplementary Figure 6. Molecular simulation shows that METTL14 R255me facilitates binding with its substrate RNA.

a-b The root mean square deviation of METTL14 (a) and RNA (b) in R255 unmethylated or mono-methylated systems.

c The radius of gyration in R255 unmethylated or mono-methylated system.

d The solvent accessible surface area in R255 unmethylated or mono-methylated system.

e The root mean square fluctuation (RMSF) of RNA bases GGACU in R255 unmethylated or mono-methylated system.

f The number of hydrogen bonds formed between RNA and R255 unmethylated or mono-methylated METTL14 during molecular simulation.

g The binding energy of RNA with R255 unmethylated or mono-methylated METTL14 during molecular simulation. For a-g, data from three simulation replicates was shown, and Source data are provided in a Source Data file.

h The binding pattern of RNA on the surface of R255 unmethylated or mono-methylated METTL14. RNA is represented as a ball-and-stick model. The differences are indicated by red dashed circles.

i The binding pattern of RNA with R255 unmethylated or mono-methylated METTL14 after 30 ns or 50 ns simulation. RNA and the asscociated amino acids are shown as ball-and-stick models. Dashed green lines represent hydrogen bonds. Amino acids of METTL3 and METTL14 are shown in black and blue, respectively. The orange letters represent the RNA.

Supplementary Figure 7. Characterizations of R255K mESCs.

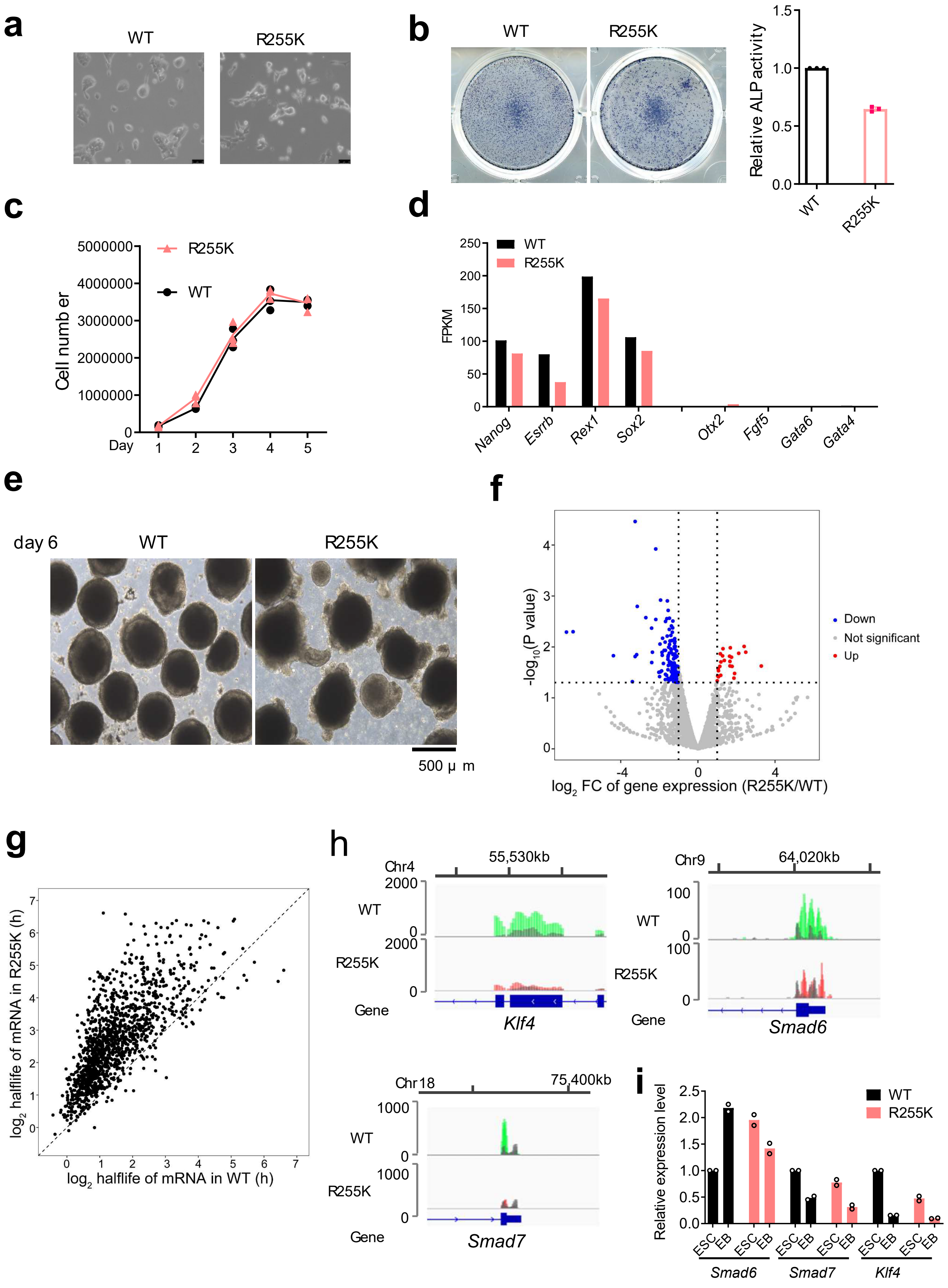

Supplementary Fig. 7. Characterizations of R255K mESCs.

a Phase-contrast microscopy showing clone morphology of WT and METTL14 R255K cell lines. Scale bar, 50μm. Representative figures of three independent replicates are shown.

b Alkaline phosphatase (ALP) staining of WT and R255K cell lines. The ALP activity of R255K was normalized to WT. Representative figures of three independent replicates are shown.

c Growth curve of cell proliferation kinetics of WT and R255K cell lines. Data from three independent experiments are shown.

d Expression level of pluripotency markers and differentiation markers in WT and R255K mESCs.

e Embryoid body (EB) morphology of WT and R255K cell lines after differentiating for 6 days. Representative figures of three independent replicates are shown.

f Differential gene expression analysis of m^6A down-regulated genes by DESeq2.

g Half-life of m^6A down-regulated genes in WT and METTL14 R255K cells.

h Profile of MeRIP and input of m^6A peaks on *Klf4*, *Smad6,* and *Smad7* in WT and R255K cell lines. Shown are normalized density of IP (green or red) versus input (gray). Values on the left show the range of IP and input density.

i Relative expression level of *Smad6, Smad7,* and *Klf4* in WT and R255K ESCs or EBs. Data are mean ± s.d. from two independent experiments. For b-d, and i, Source data are provided in a Source Data file.

Supplementary Tables

Supplementary Table 1

List of siRNAs used in this study.

#	siRNA Name	SEQUENCE
1	NC	CGUGAUUGCGAGACUCUGA
2	siRNA Prmt1-1	GCCTGCAAGTGAAGCGGAA
3	siRNA Prmt1-2	CGTCAAAGCCAACAAGTTA

Supplementary Table 2

List of primers used in this study

#	Primer Name	SEQUENCE	Primer Purpose
1	mGapdh-F	GCGAGACCCCACTAACATCAAATG	qPCR
2	mGapdh-R	GTGGTTCACACCCATCACAAACAT	qPCR
3	mNedd4-F	ACCCCAGAAGTTCATGTTTTCAC	RIP-qPCR
4	mNedd4-R	TTGCTTCTGCAGAGCGTTTGGGA	RIP-qPCR
5	mZfp36l1-F	TCCCTCCCTACCCTGGCTTAGTCA	RIP-qPCR
6	mZfp36l1-R	TCAGGATTCTCTCTCGGACCACGA	RIP-qPCR
7	mGas213-F	CACCCTGATTTGCTGAAACGATA	RIP-qPCR
8	mGas213-R	GCAGACAGCGGAGATAAAATGC	RIP-qPCR
9	mTrim71-F	TCTTGCCCTCAGAATTCACTGCT	RIP-qPCR
10	mTrim71-R	CAGGATCATAGTGGCCGACAAAG	RIP-qPCR
11	hMETTL14-up	ATGGATAGCCGCTTGCAGGAGA	Gene cloning
12	hMETTL14-dn	TCGAGCCCGGGGGATCCTTATCGAGGTGGAAAGCCACCTC	Gene cloning
13	hMETTL14R255K-up	TGGGGTTACAGAAGATGTGAAGATATTTGTTGGATTAAAACC	Gene cloning

14	hMETTL14R255K-dn	TATCTTCACATCTTCTGTAACCCCATTTTCGTAAACACACTCTTC	Gene cloning
15	s-f	CCTGCAGGATATCATCGATGGATCCAAAGAAACCGCTGCTGCT	Tag cloning
16	hMETTL14 NLS-dn	TCTCCTGCAAGCGGCTATCCATAGGCGCGTCAGCGC	Gene cloning
17	hMETTL14 MTD-up	CCTAAACTGAGGGAGCTCATCAGGC	Gene cloning
18	hMETTL14 MTD-dn	GCCTGATGAGCTCCCTCAGTTTAGGCATAGGCGCGTCAGCGC	Gene cloning
19	hMETTL14 RGG-up	CGACCAAAATCGCCTCCTCC	Gene cloning
20	GFP-up	AATTCATGGTGAGCAAGGGCGA	Gene cloning
21	GFP-dn	TCGAGCCCGGGGGATCCACTTGTACAGCTCGTCCATGC	Gene cloning
22	mActb-F	GGCTGTATTCCCCTCCATCG	qPCR
23	mActb-R	CCAGTTGGTAACAATGCCATGT	qPCR
24	mTecr-F	TACTGACTTCTCTCTGTAGAGGGGA	qPCR
25	mTecr-R	AGCTCACCGTCACCCAGCTGATCT	qPCR
26	mAnkrd9-F	CTCAGCCAAGGATAAGGGTGAG	qPCR
27	mAnkrd9-R	GCTTGGTCATTAGAGTCCCCA	qPCR
28	mThap2-F	GAAGGTGGATCAAAGCCACG	qPCR
29	mThap2-R	CTGTTCTGAGATGCCCTTAGG	qPCR
30	mZbtb41-F	GGTCTCTGCTCCTTGGGAAAA	qPCR
31	mZbtb41-R	GGGCACTTCCGATTCCTCTT	qPCR
32	hGAPDH-F	TCTATAAATTGAGCCCGCAGC	qPCR
33	hGAPDH-R	CCAATACGACCAAATCCGTTG	qPCR
34	hPRMT1-F	ACGCTGAGGACATGACATCCAAAG	qPCR
35	hPRMT1-R	TTGTCCTTGAAGAGGTGCCGGTTA	qPCR

36	mSox17-F	GCCAAAGACGAACGCAAGCG	qPCR
37	mSox17-R	TTCTCTGCCAAGGTCAACGCCT	qPCR
38	mGata6-F	CTTGCGGGCTCTATATGAAACTCCAT	qPCR
39	mGata6-R	TAGAAGAAGAGGAAGTAGGAGTCATAGGG ACA	qPCR
40	mPitx2-F	CAGAGGACTCATTTCACTAGCC	qPCR
41	mPitx2-R	CGGCGATTCTTGAACCAAAC	qPCR
42	mPdgfra-F	CAACGTCAGAACTGAATCTGGAGAT	qPCR
43	mPdgfra-R	CACTTCTCCAGGGTAAGTCCACTGC	qPCR
44	mBrachyury-F	CTGTGACTGCCTACCAGAATGAGGAG	qPCR
45	mBrachyury-R	GGTCGTTTCTTTCTTTGGCATCAAG	qPCR
46	mFlk1-F	GCTTGCTCCTTCCTCATCTC	qPCR
47	mFlk1-R	CCATCAGGAAGCCACAAAGC	qPCR
48	mBmp5-F	GGAGGCTTGGGAGACAATCACGTTC	qPCR
49	mBmp5-R	GGCAGAAGATGCTTGTTTCCCTGGT	qPCR
50	mFgf8-F	ATGGCAGAAGACGGAGACCCCTT	qPCR
51	mFgf8-R	GGCAATTAGCTTCCCCTTCTTGT	qPCR
52	mEomes-F	TGCAAGAGAAAGCGCCTGTCTC	qPCR
53	mEomes-R	CAATCCAGCACCTTGAACGACC	qPCR
54	mPax6-F	CGGGACTTCAGTACCAGGG	qPCR
55	mPax6-R	CTTCATCCGAGTCTTCTCCG	qPCR
56	mEfnb2-F	AGGAATCACGGTCCAACAAG	qPCR
57	mEfnb2-R	GAACCTGGATTTGGCTTCAC	qPCR
58	mNestin-F	CTCTTCCCCCTTGCCTAATACC	qPCR
59	mNestin-R	TTTAGGATAGGGAGCCTCAGACAT	qPCR
60	mGSC-F	TCCAGGAGACGAAGTACCCAGACGT	qPCR
61	mGSC-R	CTCGGCGGTTCTTAAACCAGACCT	qPCR
62	mFoxa2-F	CCCTACGCCAACATGAACTCG	qPCR
63	mFoxa2-R	GTTCTGCCGGTAGAAAGGGA	qPCR

64	mKlf4-F	TGCCAGAAGTGTGACAGGGCCTTTT	RIP-qPCR
65	mKlf4-R	TCCCCTCGTGGGAAGACAGTGTGAA	RIP-qPCR
66	mSmad6-F	TCGGGACTCCACAGCCTCCAACA	RIP-qPCR
67	mSmad6-R	TCGCGACTGCTGCTTCTGGAGCA	RIP-qPCR
68	mSmad7-F	AGGCTCTACTGTGTCCAAGAGCCCT	RIP-qPCR
69	mSmad7-R	ACAGCCGATCTTGCTCCGCACTTT	RIP-qPCR
70	mKlf4-F	GTTGGCGTGAGGAACTCTCTCACAT	qPCR
71	mKlf4-R	GTTGGAAAGGATAAAGTCTAGGTCC	qPCR
72	mSmad6-F	ACGGTGTGTTGCAACCCCTACCAC	qPCR
73	mSmad6-R	GACAATGTAGAATCGGACAGATCC	qPCR
74	mSmad7-F	CAACTTCTTCTGGAGCCTGGGGAT	qPCR
75	mSmad7-R	TTGAGCTGTCCGAGGCAAAAGCCA	qPCR
76	Mettl14R255K-F	CGCAAATGGGGATACAGAAAATGTGAAG	Gene cloning
77	Mettl14R255K--R	TATCCCCATTTGCGCAAGCACTAGAAAT	Gene cloning

Supplementary Table 3

List of sgRNAs used in this study.

#	sgRNA Name	SEQUENCE
1	sgRNA Mettl14 R255	ATTTCTAGTGCTTGCGAAAG
2	sgRNA Prmt1-1	CATACAAGGTGGCCCGGTCT
3	sgRNA Prmt1-2	CGACTACGTGCACGCGCTGG

Supplementary Table 4

KEGG pathway enrichment analysis of differentially expressed genes with R255 methylation regulated m^{6}A peaks in WT and METTL14 R255K cells. One-sided Hypergeometric test, FDR was corrected with Benjamini and Hochberg.

Term	P-Value	FDR	Genes
Herpes simplex virus 1 infection	3.16E-06	9.13E-05	*Zfp617|Zfp229|Zfp760|Zfp961|Zfp850|Zfp345|Zfp619|Zfp709|Zfp871|Zfp101|Zfp386|Zfp780b*
Viral carcinogenesis	0.006776	0.05128	*Rbpj|D1Pas1|Ube3a|Rb1|Creb1*
