# Supplementary material for: Arginine methylation of METTL14 promotes RNA N6-methyladenosine modification and endoderm differentiation of mouse embryonic stem cells
Source: Nat Commun. 2021 Jun 18;12:3780. doi: 10.1038/s41467-021-24035-6 (PMC8213825; doi:10.1038/s41467-021-24035-6)

Fig.1

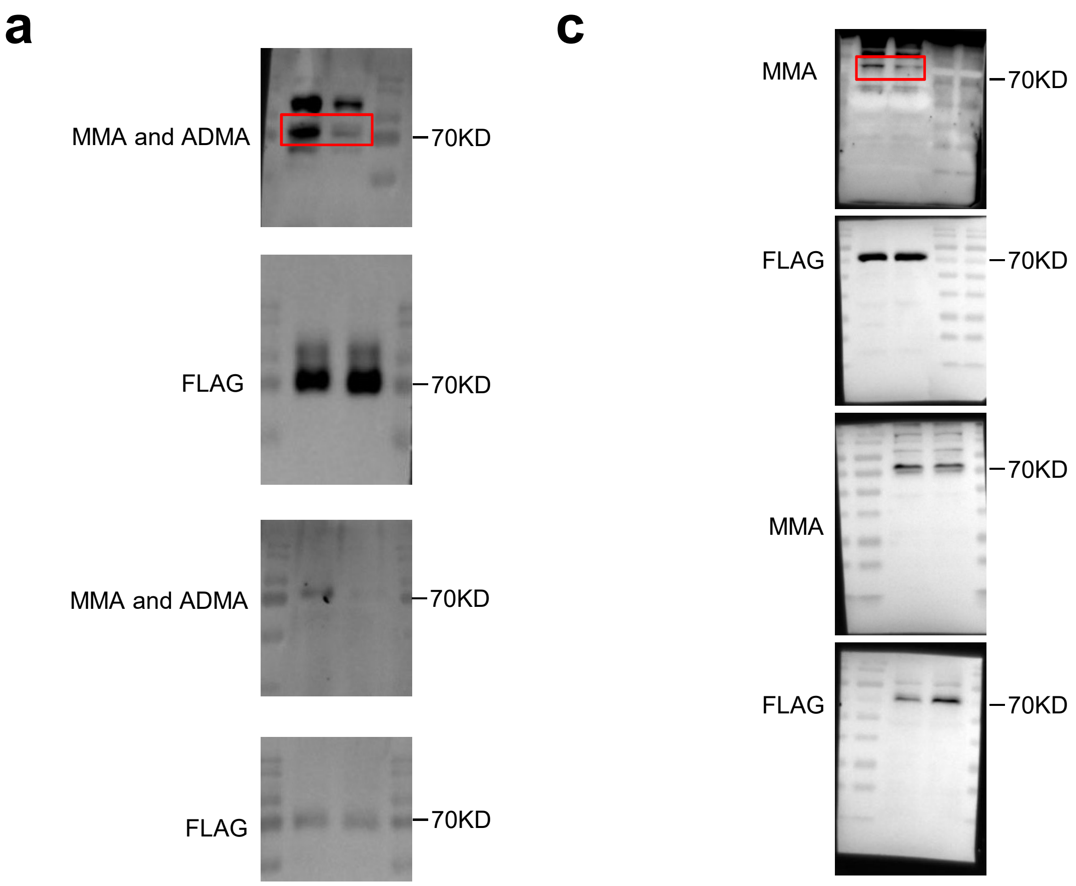
a
MMA and ADMA
70KD
FLAG
70KD
MMA and ADMA
70KD
FLAG
70KD
c
MMA
70KD
FLAG
70KD
MMA
70KD
FLAG
70KD

Fig.2

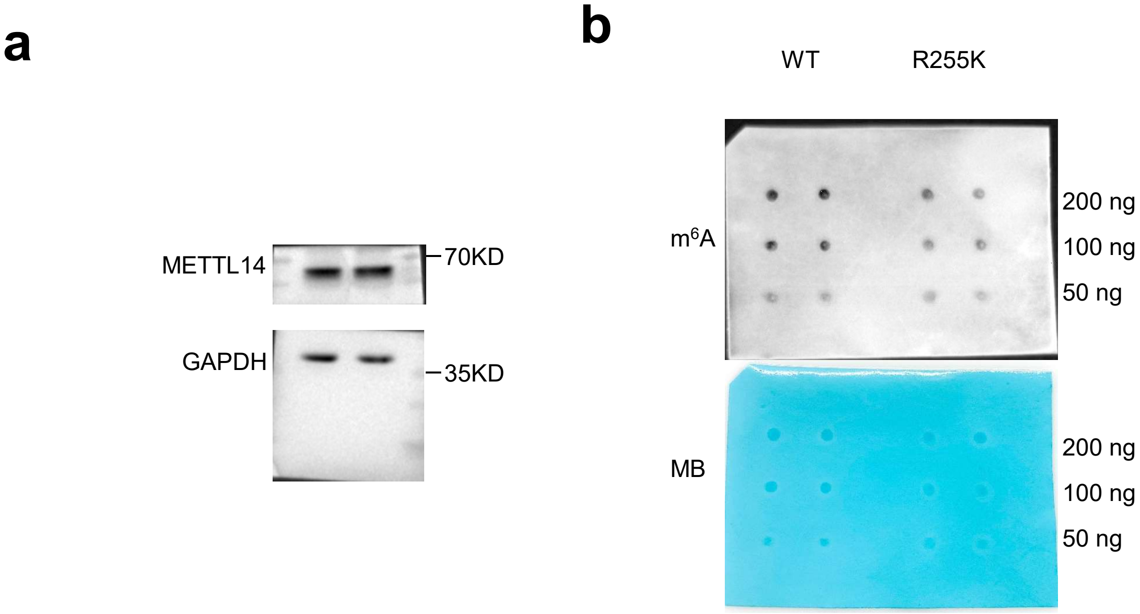

a
METTL14
70KD
GAPDH
35KD
b
WT
R255K
m6A
200 ng
100 ng
50 ng
MB
200 ng
100 ng
50 ng

Fig.3

b

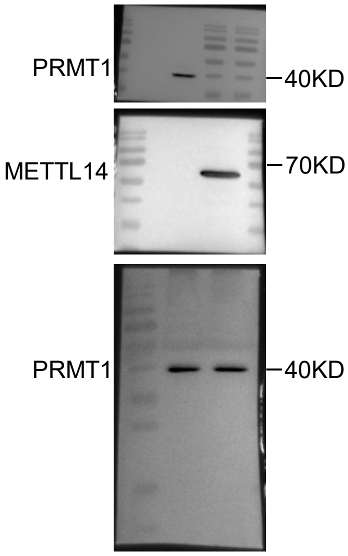
PRMT1
40KD
METTL14
70KD
PRMT1
40KD

c

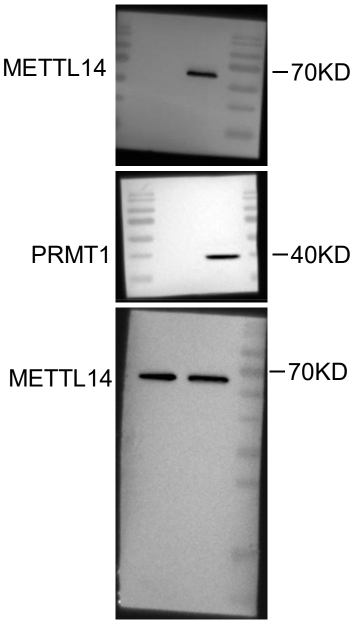
METTL14
70KD
PRMT1
40KD
METTL14
70KD

e

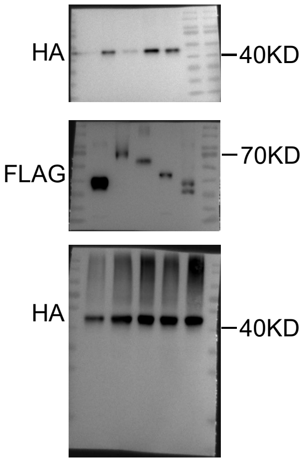
HA
40KD
FLAG
70KD
HA
40KD

f

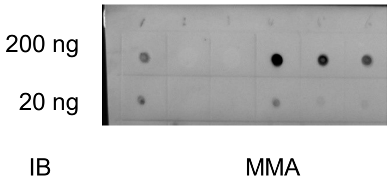
200 ng
20 ng
IB
MMA

Fig.5

a

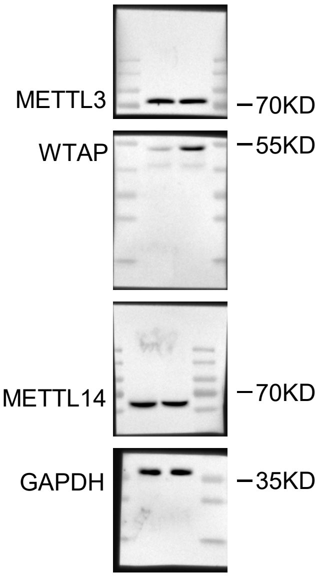
METTL3
70KD
WTAP
55KD
METTL14
70KD
GAPDH
35KD

b

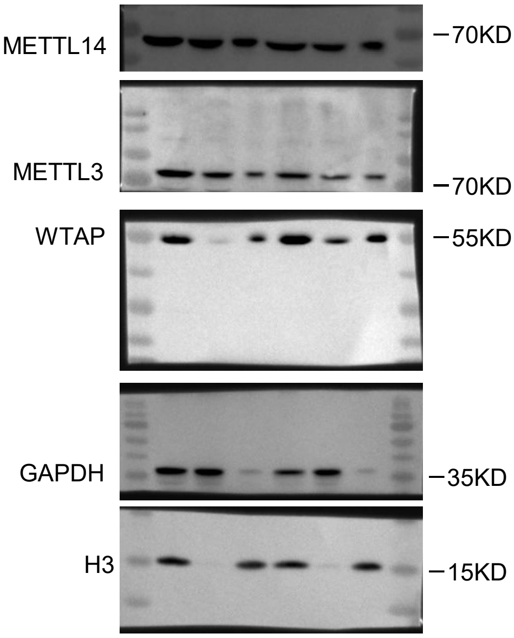
METTL14
70KD
METTL3
70KD
WTAP
55KD
GAPDH
35KD
H3
15KD

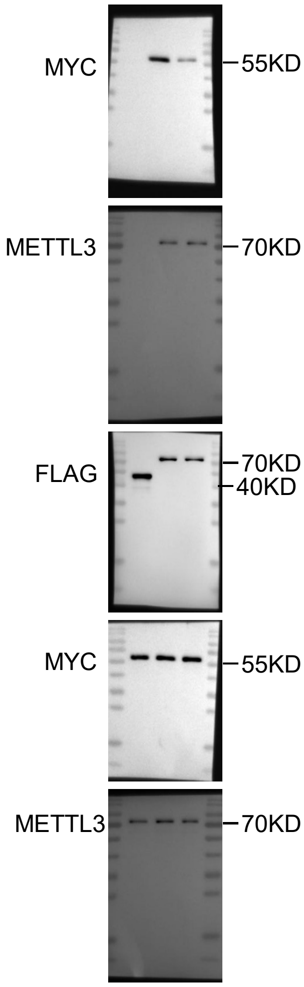
MYC
55KD
METTL3
70KD
FLAG
70KD
40KD
MYC
55KD
METTL3
70KD

c

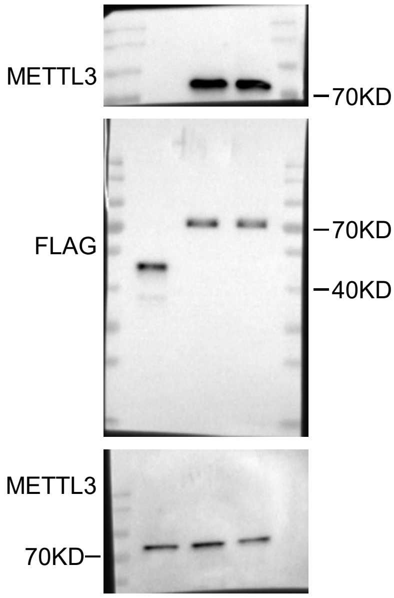
METTL3
70KD
FLAG
70KD
40KD
METTL3
70KD

f

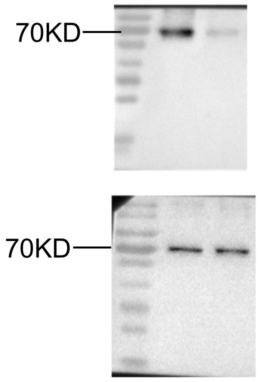
70KD
70KD

Supplementary Fig 1

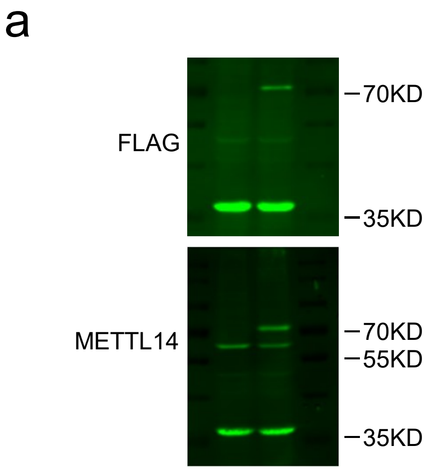

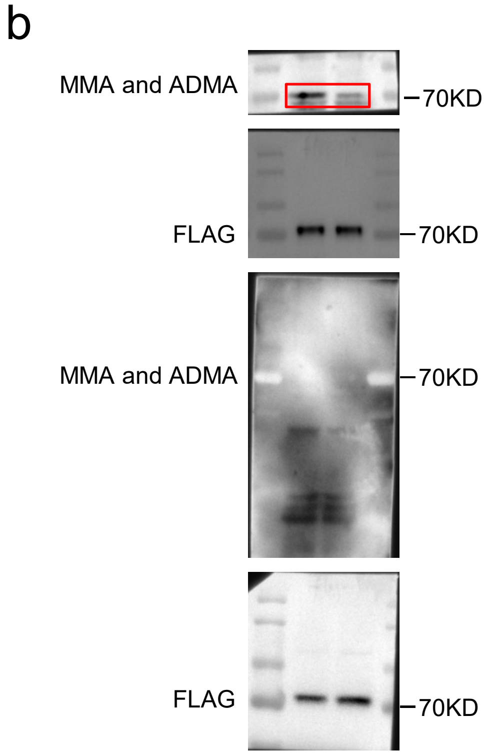

Supplementary Fig 2

d

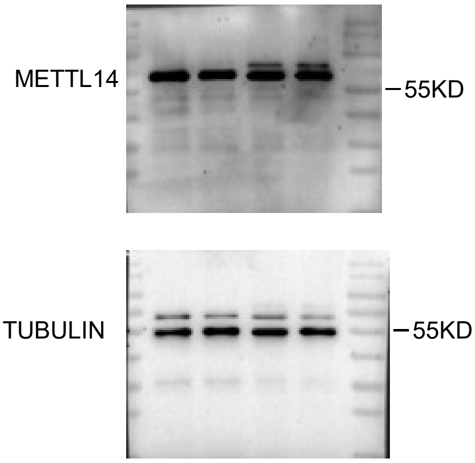

Supplementary Figure 3

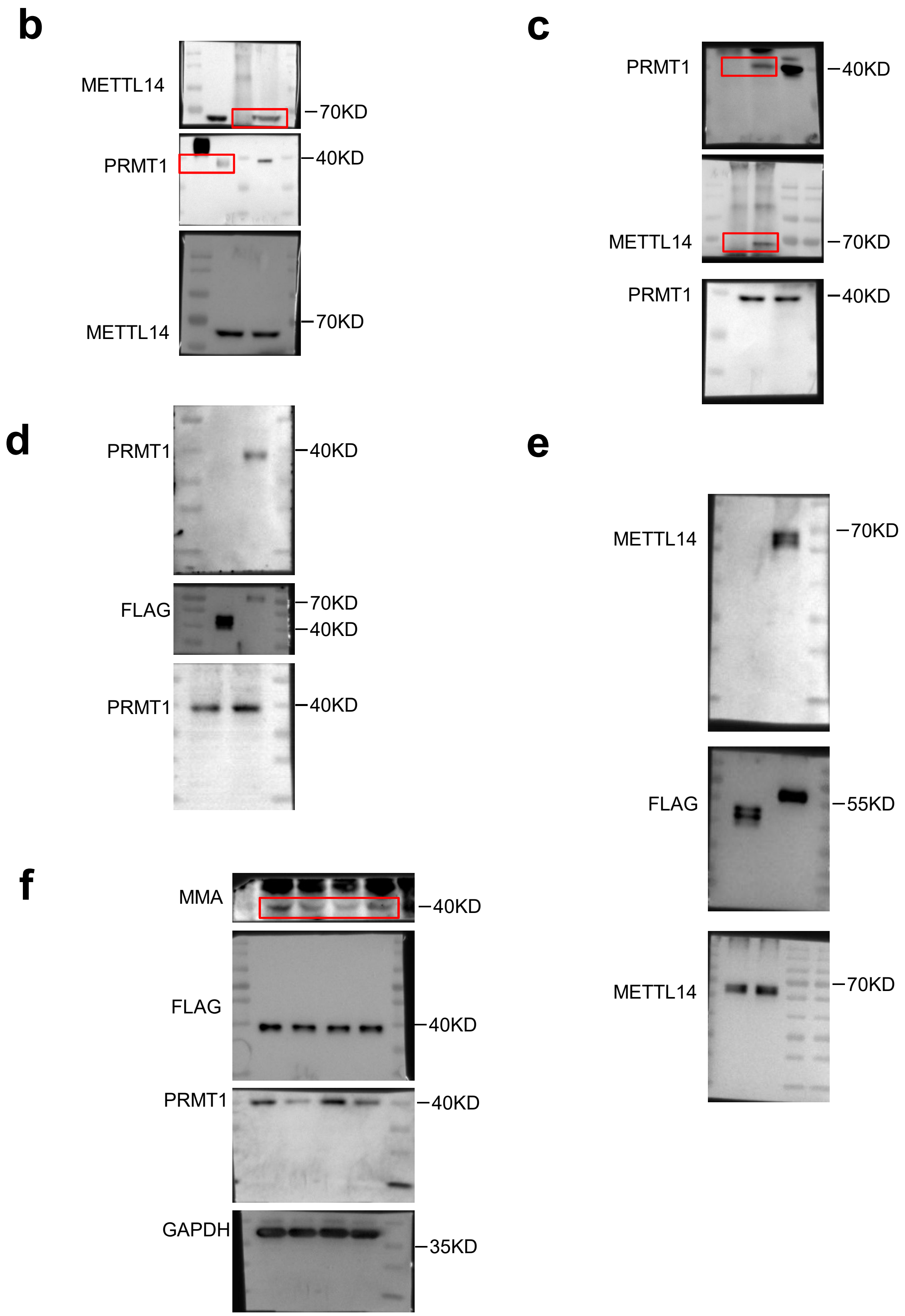

Supplementary Figure 4

a

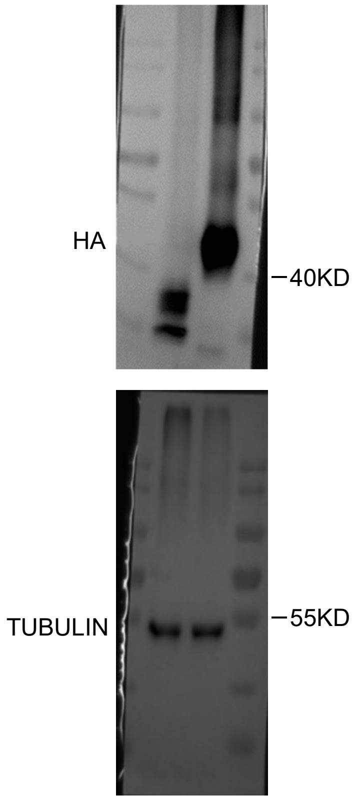

b

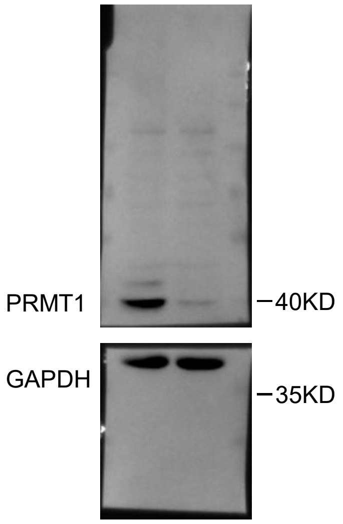

d

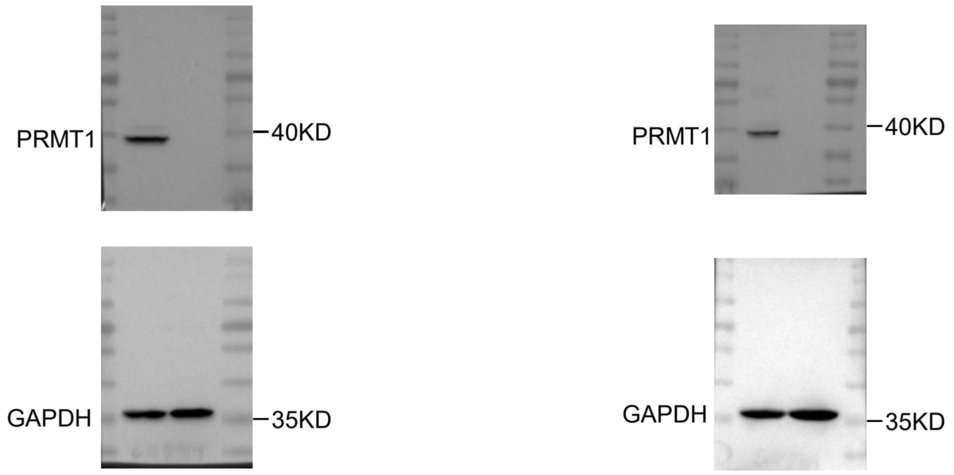

f

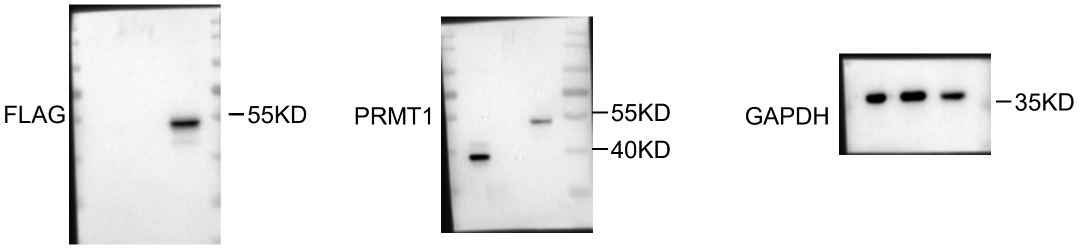

Supplementary Fig.5

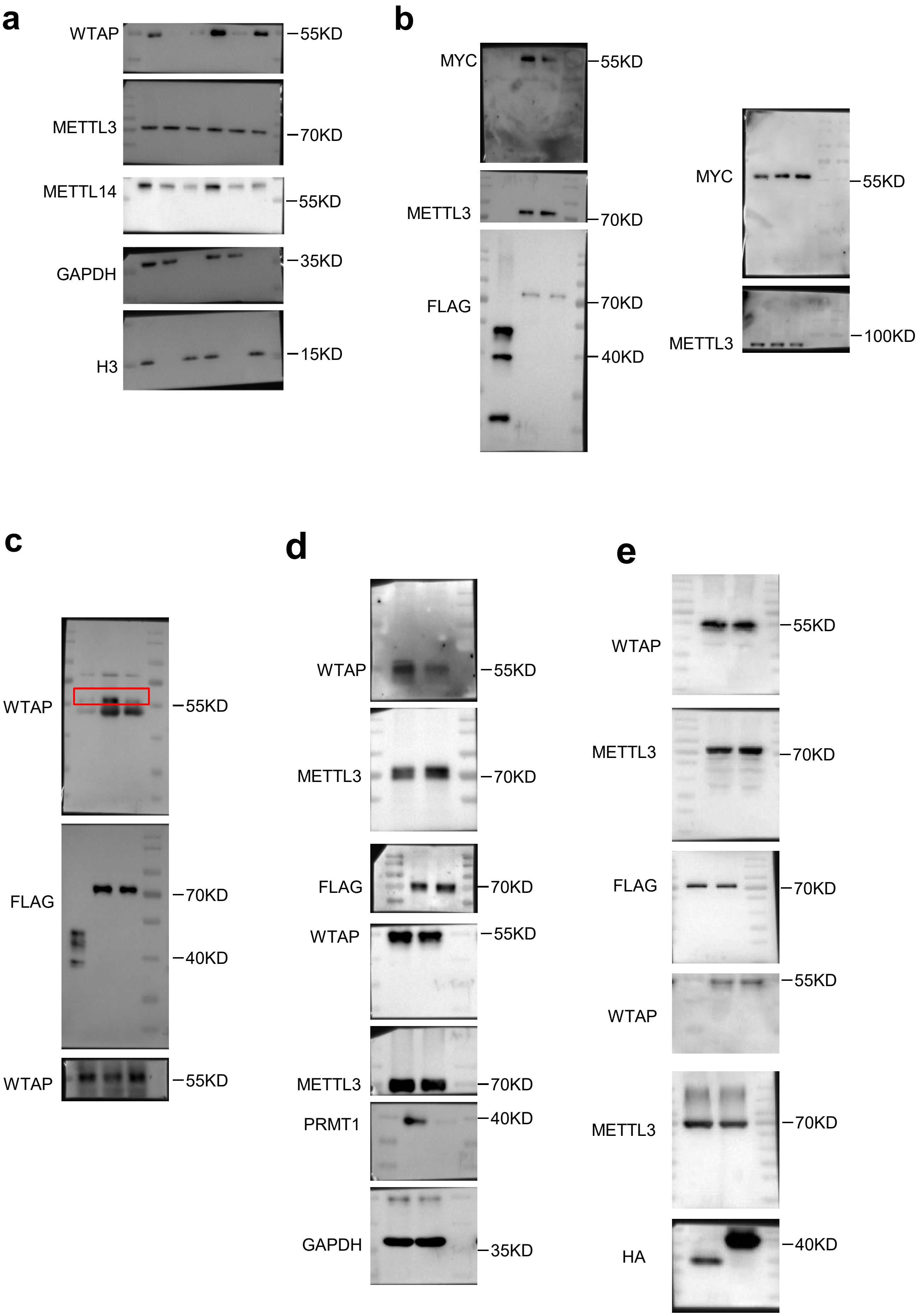

Supplement: Supplementary file 4 — Source Data [file 41467_2021_24035_MOESM4_ESM.zip › uncropped blots.pdf]
